# Pediatric bronchogenic cysts in the head and neck region: A study of 10 surgical cases and a review of the literature

**DOI:** 10.3389/fped.2022.1030692

**Published:** 2022-11-03

**Authors:** Wei Chen, MengRou Xu, Qingyu Wang, Hongming Xu, Jiarui Chen, Xiaoyan Li

**Affiliations:** ^1^ Department of Otolaryngology-Head and Neck Surgery, Shanghai Children's Hospital, School of Medicine, Shanghai Jiao Tong University, Shanghai, China; ^2^ Department of Pathology, Shanghai Children's Hospital, School of Medicine, Shanghai Jiao Tong University, Shanghai, China

**Keywords:** bronchogenic cysts, children, head, neck, surgery

## Abstract

**Objective:**

To explore the clinical characteristics and surgical treatment of children with bronchogenic cysts (BCs) in the head and neck region.

**Methods:**

A retrospective study of 10 pediatric patients with BCs in the head and neck region treated in Shanghai Children's Hospital during 2011 to 2022 was performed.

**Results:**

Based on their pathological diagnosis, 10 patients with BCs in the head and neck were identified. The most common location was the neck (8 patients, 80%; 2 midline neck, 6 lateral neck), followed by the ventral tip of tongue (1 patient), and the posterior pharyngeal wall (1 patient). Misdiagnosed as lymphangioma in 5 cases, cyst in 3 cases, thyroglossal duct cyst (TGDC) in 2 cases and congenital pyriform sinus fistula (CPSF) in 1 case preoperative. The median follow-up period after surgery was 4.68 (range, 0.67–9.25) years. All 10 patients underwent complete resection without recurrence or other complications.

**Conclusions:**

Although extremely rare, BCs should be considered in the differential diagnosis of midline and lateral neck masses or intraoral cysts in children. Surgical excision is recommended in BCs, and the diagnosis is definitively confirmed by histopathology.

## Introduction

Bronchogenic cysts (BCs) are thought to be formed by small buds of diverticula that separate from the foregut during formation of the tracheobronchial tree (1). BCs are rare congenital diseases with an incidence rate of 1/42,000–68,000 (2). According to the location of the lesion, they can be divided into three types: intrapulmonary type, mediastinal type and ectopic type.

More than 99% of the patients occur in the mediastinum and lungs, while less than 1% affect in the head and neck (1, 3). Preoperative diagnosis of BCs in the head and neck region remain challenging (3). The location of a bronchogenic cyst (BC) varies greatly. Because of its rarity, it is often misdiagnosed as a more common congenital neck cyst (3, 4). Herein, we retrospectively analyzed the clinical features and surgical treatment results of children with head and neck BCs.

## Materials and methods

In the present study, we performed a retrospective review of 10 patients presenting with cystic masses in the head and neck region who were histopathological diagnosed with BCs between January 2011 to January 2022. The children were identified from the Department of Otolaryngology Head and Neck Surgery at Shanghai Children's Hospital. Cases were assessed by parameters including age at diagnosis, gender, clinical symptoms, symptoms duration, misdiagnosis history, infection and treatment history, location and size of the BCs, diagnostic methods, treatment method, operative notes, postoperative complications, histopathological diagnosis, length of the follow-up, and recurrence. All patients with BCs received surgical treatment. This study was approved by our institutional Research Ethics Board (Approval No: 2022R067-E01).

## Results

**Figure 1:**
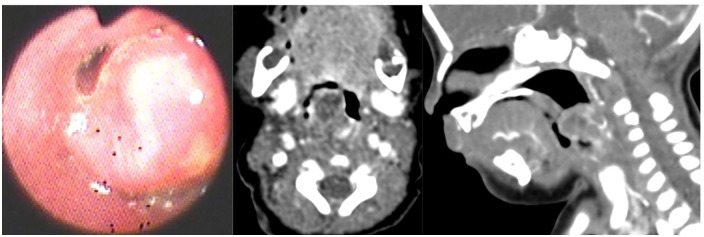
A 19-hour-old female. (**A**) Fiberoptic laryngoscopy showed a large globular mass (white arrow) with a smooth mucosal surface in the posterior pharyngeal wall. CT revealed a 1.4-cm sized cystic lesion in the posterior pharyngeal wall (red circle): (**B**) axial contrast-enhanced CT; (**C**) sagittal contrast-enhanced CT.

**Figure 2:**
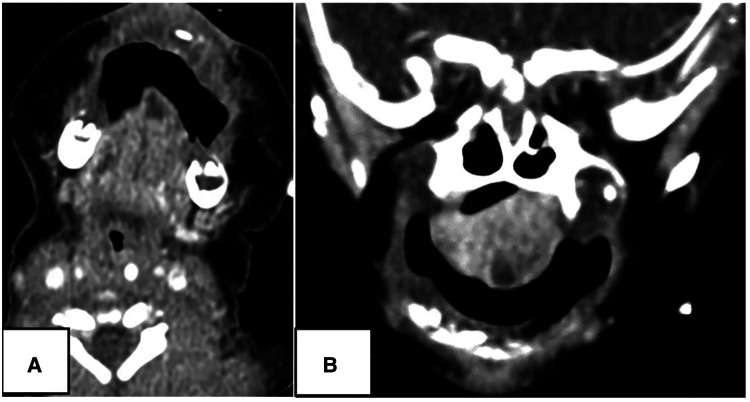
A 6-hour-old Male. CT showed a 1.0-cm sized cystic lesion in the ventral tip of tongue (white arrow): (**A**) contrast-enhanced axial scan; (**B**) coronal scan.

There were 10 patients (5 males and 5 females; age range, 6 h–5 years; median, 2.25 years) in this study (Table 1). Seven patients presented with asymptomatic masses in the neck. One patient (1.8-year-old female) had a history of recurrent infection in the midline neck, and had undergone incision and drainage. One newborn showed stridor after birth, and fiberoptic laryngoscopy revealed a mass in the posterior pharyngeal wall (Figure 1). The remaining 1 patient was incidentally diagnosed by routine physical examination (Figure 2). The duration of symptoms was 6 h to 5years (median, 8 months). Misdiagnosed as lymphangioma in 5 cases, cyst in 3 cases, TGDC in 2 cases and CPSF in 1 case before operation.

**Table 1:** Clinical summary of head and neck BCs.

Patient	Age	Sex	Symptom/Duration	Location	Misdiagnosis	Diagnosis	Size	Surgery	Recurrence	FU
1	3 year	M	Mass/12 month	R: neck	Lymphangioma	CT	2.1 cm	External	No	9.25 year
2	6 h	M	Mass/6 h	Ventral tip of tongue	Sublingual cyst	CT	1.0 cm	Intraoral	No	5.58 year
3	19 h	F	Stridor/19 h	Posterior pharyngeal wall	Cyst	CT, Laryngo	1.4 cm	Endoscopic	No	5.42 year
4	2 year	F	Mass/6 month	L: neck	Lymphangioma, CPSF	CT, US	3.4 cm	External	No	4.75 year
5	1 year	M	Mass/2 month	Midline neck	TGDC	CT, US, Laryngo	2.3 cm	External	No	6.75 year
6	5 year	F	Mass/5 year	L: neck	Lymphangioma	CT, US	7.5 cm	External	No	4.60 year
7	2.5 year	M	Mass/8 month	R: neck	Cervical cyst	CT, US, Laryngo	4.9 cm	External	No	4.58 year
8	1.8 year	F	Mass/9 month	Midline neck	TGDC	CT, US, Laryngo	2.1 cm	External	No	4.50 year
9	3.3 year	M	Mass/2 year	L: neck	Lymphangioma	CT, US, Laryngo	2.5 cm	External	No	4.33 year
10	2.9 year	F	Mass/8 month	R: neck	Lymphangioma	CT, US, MRI	4.2 cm	External	No	8 month

BCs, bronchogenic cysts; CT, computed tomography; US, ultrasonography; MRI, magnetic resonance imaging; F, female; M, male; L: left; R: right; h, hour; mo, month; y, year; cm, centimeter; CPSF, congenital pyriform sinus fistula; TGDC, thyroglossal duct cyst; Laryngo, fiberoptic laryngoscopy; FU, follow-up.

The most common location of BCs was the neck (8 cases, 80%; 2 midline neck, 6 lateral neck), followed by the ventral tip of tongue (1 case, 10%), and the posterior pharyngeal wall (1 case, 10%). The size of the cysts ranged from 1 to 7.5 (median, 2.3) cm. Contrast enhanced computed tomography (CT) was performed in 10 cases, ultrasound (US) in 7 cases, and magnetic resonance imaging (MRI) in 1 case to determine the extent of the cyst and exclude other diseases. Uncooperative patients were given 10% chloral hydrate (0.5 ml/kg) orally prior to the CT examinations.

All 10 patients with head and neck BCs received surgical treatment. Eight cases underwent external cervical approach under general anesthesia. The remaining 2 patients underwent intraoral and endoscopic surgery under general anesthesia. All patients with BCs were confirmed by histopathology.

All cases were cured without complications and no recurrence during follow-up. The median follow-up period after surgery was 4.68 (range, 0.67–9.25) years.

## Discussion

At the third week of the embryo, the foregut undergoes dichotomous development. The ventral side forms the respiratory tract, and the dorsal side forms the esophagus, duodenum and stomach. During the development of tracheobronchial trees, developmental defects and abnormal division can lead to the formation of BCs (1, 2). BCs are rare congenital malformations that usually occur in the lung parenchyma or mediastinum (4, 5). However, BCs appear in the neck are particularly rare (2, 6). Previous reports have described that the positions of BCs in the head and neck are variable, from the oral cavity to the midline and lateral neck regions (3, 7). Lee DH (3) reported that clinicians should be aware that BCs in the head and neck may exist in many locations, such as the soft palate, the posterior pharyngeal wall, the floor of the mouth, and the arytenoid cartilage. In our studies, 7 cases were located in the neck, 1 case in the tongue and 1 case in the posterior pharyngeal wall, which was consistent with the literature.

BCs in the head and neck region are usually asymptomatic cystic lesions, and most cases are found during physical examinations (8), unless they are large enough to cause compression of the surrounding tissue or secondarily infected (9). In some individuals, large cysts may cause respiratory distress, wheezing, cough, dyspnea, and dysphagia (8). If the cyst is shallow, it may lead to sinus formation and external drainage of pus. If the cyst is deep, it may lead to abscess formation (1, 10, 11). In our series, 7 cases showed asymptomatic neck mass, 1 case had secondary infection of neck mass and underwent incision and drainage. One case had stridor after birth, and a posterior pharyngeal wall mass was found by fiberoptic laryngoscopy. There was 1 case incidentally diagnosed by physical examination. The clinical symptoms in our study were consistent with the above-mentioned cyst characteristics.

Although physical examination is the most important factor in diagnosis, assistant examination can provide valuable information for the evaluation of congenital cervical masses, especially in children (5). US, CT, and MRI are helpful in the diagnosis of BCs (3, 9). US typically shows a unilocular cystic lesion filled with fluid (4).

**Figure 3:**
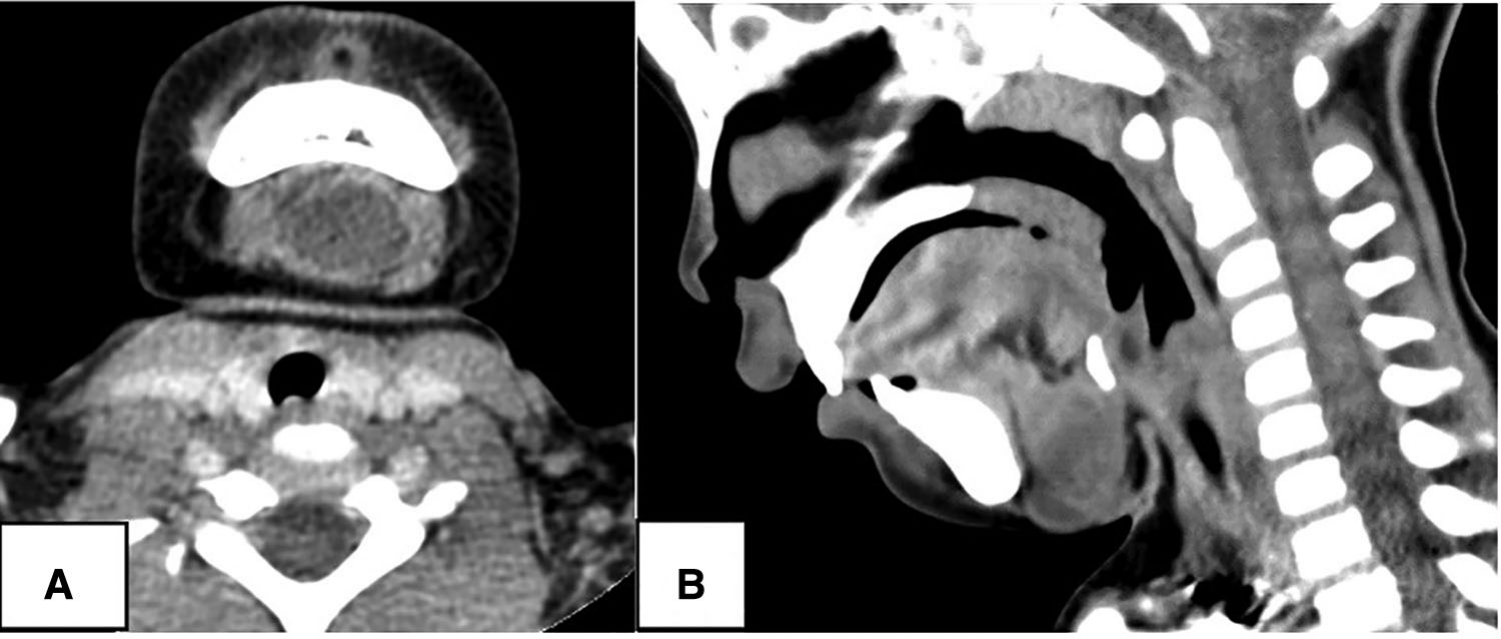
A 1.8-year-old female. CT showed a 2.1-cm sized cystic lesion in the midline neck connecting with hyoid bone (red circle), and misdiagnosed as thyroglossal duct cyst: (**A**) contrast-enhanced axial scan; (**B**) sagittal scan.

**Figure 4:**
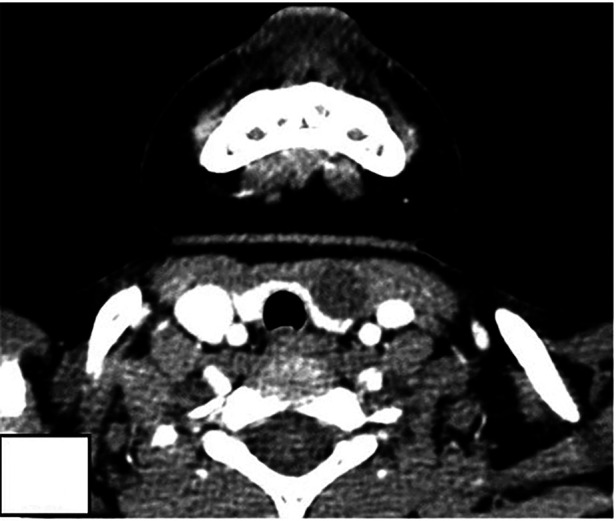
A 3.3-year-old Male. CT showed a 1.5-cm sized homogeneous, low-density mass in the left neck region (white arrow), and located anterolaterally of thyroid. CT showed that the left thyroid was compressed and deformed. This case was misdiagnosed as lymphangioma preoperative.

**Figure 5:**
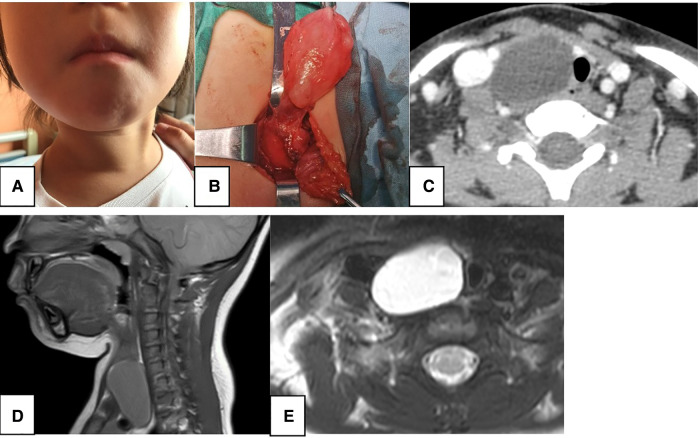
A 2.9-year-old female. CT showed a 4.2-cm sized homogeneous, low-density mass (**C**, white arrow) with thin walls and smooth borders in the right neck region without enhancement, and misdiagnosed as lymphangioma. The right thyroid was compressed and deformed, and the trachea (**C,E**, red circle) shifted to the opposite side. MRI showed low signal intensity on T1WI (**D**, white arrow) and high signal intensity on T2WI (**E**, white arrow). (**A**) preoperative neck mass; (**B**) intraoperative mass; (**C**) axial contrast-enhanced CT; (**D**) sagittal T1WI MRI; (**E**) axial T2WI MRI.

CT and MRI are more helpful to detect the extent of the lesion and determine the relationship between the lesion and the surrounding anatomical structure, which also helps to formulate the surgical strategy (3, 9, 12). CT is convenient and fast, while MRI examination takes a long time and costs high. For these reasons, we generally perform enhanced CT scans. BCs are usually round or oval lesions with smooth edges, uniform density, or air-liquid level due to repeated infection. However, there are currently no specific imaging standards for BC (13), although imaging methods are useful in the case of planning the surgery (10). In this study, a 1.8-year-old female and a 1-year-old male, our preoperative diagnosis were TGDC around the hyoid bone (Figure 3). Therefore, the location of BCs cannot be used to make a final diagnosis (3). Due to the low incidence rate, the rates of misdiagnosis and missed diagnosis are high preoperative (8). The differential diagnosis of BCs in the head and neck region contains TGDC, branchial cleft cyst, lymphangioma, dermoid cyst/epidermoid cyst, cervical thymic cyst, and thyroid cyst (3, 10). That is to say, the diagnosis of BC requires exclusion of all cystic masses in the neck. In the present study, 5 cases were misdiagnosed as lymphangioma, 3 cases as cyst, 2 cases as TGDC, and 1 case as CPSF before surgery (Figures 3–5). The head and neck lymphatic malformations (HNLMs) are usually diagnosed within 2 years old, almost half of which occur in neonates, and usually occur in the posterior triangle of the neck (14). CT findings of HNLMs were mostly poorly defined, multilocular and low-density masses (15, 16). Fluid-fluid levels can be seen in some patients with intracystic hemorrhage (17) TGDC usually occurs in the anterior midline of the neck, at the level of the hyoid bone, and connects with it (18). The first branchial cleft anomalies (CFBCAs) often presented with repeated swelling and purulence in Pochet's triangle. CT showed round, tubular or strip-shaped abnormal low-density shadow with unclear boundary in the parotid region, extending outward to the skin surface or EAC (19). The typical CT features of congenital second branchial cleft anomalies (CSBCAs) include isolated and uniform low-density cystic masses surrounded by uniformly thin, smooth walls. CSBCAs are generally located at the anteromedial border of the sternocleidomastoid muscle, posterior to the submandibular gland, and lateral to the carotid sheath (20, 21). The main clinical manifestations of CPSF are recurrent suppurative thyroiditis and neck abscess, predominantly located on the left sided (22). CT revealed the shallower or disappearing pyriform sinus, large cystic lesion with fluid and air, association or obscuration of the left superior thyroid lobe with the neck mass or abscess, tubular structures seen inside the thyroid and air-containing ducts originating from the piriform fossa (23, 24).

**Figure 6:**
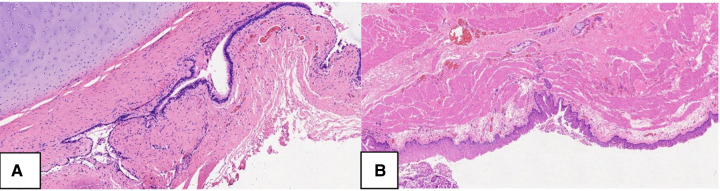
Histopathology of bronchogenic cyst. The inner layer of BC originates from the respiratory system and is covered by pseudostratified columnar ciliated epithelium on the fibrous connective tissue wall, which contains the cartilage plates, (**A**. HE, 100X), seromucous glands and smooth muscle (**B**. HE, 50X). pseudostratified columnar ciliated epithelium, PCCE; fibrous connective tissue wall, FCTW; seromucous glands, SG; smooth muscle, SM.

It was difficult to distinguish this disease from other diseases by preoperative imaging alone (8). Therefore, histopathological examination is critical to the final diagnosis of BCs (3, 4, 8, 25). The wall structure of BCs is the same as that of bronchial wall. A typical BC shows ciliated pseudostratified cubic columnar epithelium with underlying fibrous connective walls. Sometimes, variations may occur, such as the presence of cartilage, smooth muscle and seromucous glands (2, 3). The presence of cartilage is a necessary condition for the diagnosis of a BC (1). The cysts are typically filled with clear fluid, air or hemorrhagic secretions (26, 27). In this study, the postoperative pathological analysis was consistent with the above cyst characteristics of BCs (Figure 6).

Due to the risk of malignant transformation and complications, surgical resection is the most effective method to treat a BC in the head and neck (8, 9). Once a BC is suspected, surgical excision should be undertaken as soon as possible (8, 28, 29). BCs of the head and neck usually do not recur after complete resection (4). In this study, there was no recurrence or complications resulting from the surgical intervention observed.

However, this study only included a small number of patients, so more cases need to be studied in the future to confirm the study results.

## Conclusion

BCs are extremely rare congenital malformations, in addition, they can occur at various sites of the head and neck. As a result, BCs should be considered in the differential diagnosis of lateral and midline cervical masses or intraoral cysts. The definitive diagnosis, however, depends on the histopathological examination. We should perform surgical treatment as soon as possible, as surgery is the most effective method to treat a BC.

## Data Availability

The original contributions presented in the study are included in the article/Supplementary Material, further inquiries can be directed to the corresponding author/s.
